# Translating COVID-19 Pandemic Surge Theory to Practice in the Emergency Department: How to Expand Structure

**DOI:** 10.1017/dmp.2020.57

**Published:** 2020-03-27

**Authors:** Matteo Paganini, Andrea Conti, Eric Weinstein, Francesco Della Corte, Luca Ragazzoni

**Affiliations:** CRIMEDIM – Research Center in Emergency and Disaster Medicine, Università del Piemonte Orientale, Novara, Italy

**Keywords:** COVID-19, pandemics, SARS-CoV-2, surge capacity, translational science

## Abstract

Multiple professional societies, nongovernment and government agencies have studied the science of sudden onset disaster mass casualty incidents to create and promote surge response guidelines. The COVID-19 pandemic has presented the health-care system with challenges that have limited science to guide the staff, stuff, and structure surge response.

This study reviewed the available surge science literature specifically to guide an emergency department’s surge structural response using a translational science approach to answer the question: How does the concept of sudden onset mass casualty incident surge capability apply to the process to expand COVID-19 pandemic surge structure response?

The available surge structural science literature was reviewed to determine the application to a pandemic response. The on-line ahead of print and print COVID-19 scientific publications, as well as gray literature were studied to learn the best available COVID-19 surge structural response science. A checklist was created to guide the emergency department team’s COVID-19 surge structural response.

During the 20th century, health care has radically evolved due to new discoveries and changes in global politics, while the percentage of population that gained access to health care has dramatically increased. Despite this achievement, governments strain to fulfill the overwhelming request for medical assistance. Each government deploys resources trying to meet United Nations’ Sustainable Development Goal No. 3 (Ensure healthy lives and promote well-being for all at all ages)^1^ while balancing health-care expenditure within their gross domestic product.^2^ Health-care systems oscillate between 2 key elements: demand and available resources. Similar to living organisms, health care has complex mechanisms to achieve and maintain a “stability of the internal environment” (*milieu intérieur*), despite external stressors.^3^ The resulting equilibrium could resemble cellular homeostasis during normal conditions.^4^ However, extraordinary events can result in a significant imbalance.

The novel coronavirus (SARS-CoV-2) pandemic is currently threatening several national health systems.^5^ To date, Italy has been one of the most affected countries^6^ where public health departments, emergency medical systems, and hospitals are struggling to deal with the surge of patients affected by 2019-nCoV.

Unfortunately, contagion rates are estimated to rise exponentially in many countries, regardless of their health-care delivery system. Urgent actions are required to modify both health-care systems configurations and hospitals’ capacity and capability to respond. In such situations, high-quality data are limited. The application of translational science in this disaster medicine setting can provide stakeholders and clinicians with acceptable evidence-based medicine concepts.^7-9^

- **T0 = Identification of opportunities and approaches to a health problem.** How does the concept of sudden onset mass casualty incident (MCI) surge capability apply to the process to expand COVID-19 pandemic surge structure response? Structure refers to the physical location of the space where providers attend to the patient.
- **T1 = Basic research for clinical effect and/or applicability, human physiology knowledge, and potential for intervention.** Review prior MCI surge literature for specific references to surge structure reconfiguration guidance.
- **T2 = New interventions to form basis for clinical application and evidence-based guidelines.** Scoping Literature Review of available COVID-19 peer review and gray literature.^10^
- **T3 = Implementation of research findings in clinical practice.** Creation of a checklist for COVID-19 Surge Structure Planners.
- **T4 = Effects on practice influencing populations and policy.** Collection of completed checklists to improve the COVID-19 Surge Structure Planning.

## THE T0 QUESTION

A marked increase in demand for medical resources, known as “surge,”^11^ can have detrimental effects on health-care systems if the supply of available resources to meet this demand, known as surge capacity, is not sufficient.^12^ Surge capability is how surge capacity is used to meet the unexpected surge.^11^ With this perspective, the concept of resilience is more appropriate to represent the properties of health-care systems during disasters or MCIs. Initially, this engineering concept described the elastic deformation of materials under physical strain.^13^ This term was implemented in sociology as the ability of groups or communities to cope with external stresses and disturbances.^14^ Resilience can be applied to public health to illustrate the adaptations at individual, community, and system levels.^15^ A resilient health-care system must be able to limit and cope with stressors and events (absorptive capacity), to adapt itself toward external events (adaptive capacity), to forecast events and take action to minimize effects (anticipatory capacity), and to change the structures and operations to better address results (transformative capacity).^15^ Altogether, the improvement of surge capability and resilience are critical steps of disaster mitigation and preparedness, to achieve an adequate response toward sudden and high-impact events, such as disasters or outbreaks.^16^

## THE T1 REVIEW

### Mass Casualty Incidents

After the Oklahoma City Bombing in 1995,^17^ there was renewed interest in MCI management, specifically to create plans to meet the demand of a sudden supply of patients after a sudden onset terrorist disaster. Emergency Departments (EDs) would have to be able to rapidly expand the operational staff, stuff, and structures.^18^ This study led to the terrorism MCI response to factor the resilience of the first responders and the first receivers to not become terrorist victims in addition to being able to treat the overwhelming demand of injured patients. The staff, stuff and structures would have to protect all concerned.

The MCI planning and response to chemical (Aum Shinrikyo^19^) and biologic (Anthrax^20^) terrorism added to the mitigation, preparation, and response calculus. Materials to protect staff, personal protective equipment, along with training and competencies, would have to be funded and maintained for all potentially affected staff. The Severe Acute Respiratory Syndrome (SARS) epidemic of 2002-2004^21^ moved the MCI discussion from sudden onset disaster (SOD) planning and response to the unique variables to meet the demand of a sudden onset of patients with a novel disease, or a disease caused by an agent that was not known and had no specific diagnostic test or treatment. This response entailed a new calculus to prevent transmission to Emergency Medical Services (EMS) staff or emergency department (ED) and hospital patients, visitors, and staff. The 2009 H1N1/09,^22^ Middle East Respiratory Syndrome (MERS) in 2012,^23^ and Ebola in 2013^24^ tested the mettle of governments, first responders, and receivers, as well as those health-care systems.

### Pandemic Surge Capacity

**FIGURE 1:**
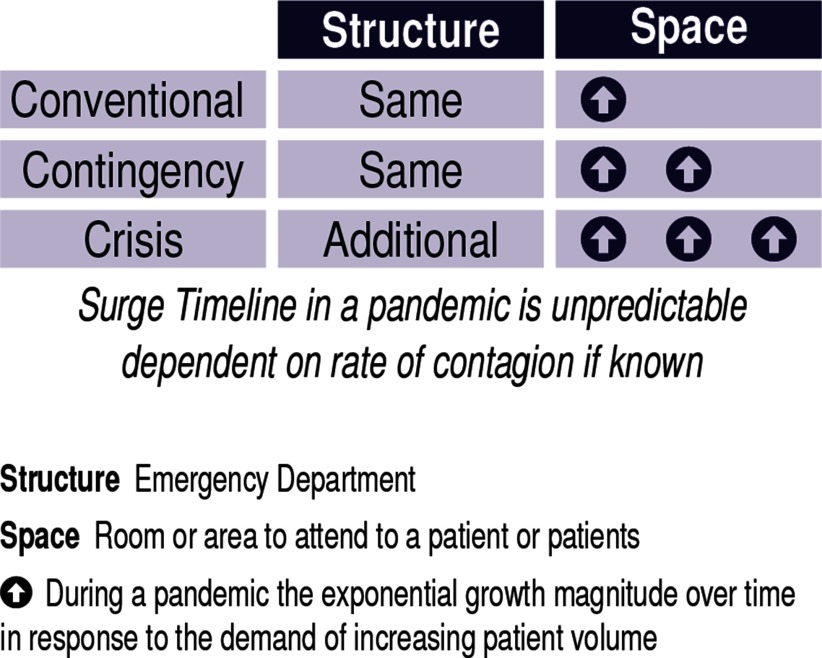
Pandemic Surge Timeline.

In 2009, planners began to adapt the structural phases in the timeline^25^ of a sudden onset MCI. Adapting this to the timeline of a COVID-19, with days to weeks before the overwhelming demand of patients present with hypoxia and respiratory failure (Figure 1).

### Conventional

When the ED is operating with typical volume or even if the ED is boarding patients, the staff can divert patients that fit the COVID-19 “person under investigation” from the entrance to the ED, bypassing triage into a room with a door or an Airborne Infection Isolation Room (AIIR) if available with standard precautions.^26^ During the time this patient or a few more present with similar symptoms and are evaluated, other typical ED patients are also arriving. The ED staff resilience features: strict efforts to not expose any ED patients and staff while creating ED space for these patients through an expedited discharge process of all other patients to designated ED areas waiting for rides; hospital admission process, including abbreviated reports or orders to the in-patients units; and other creative efforts, such as moving patients to the hallways that typically are not used when the ED is overcrowded. The ED structure adapts to meet the demand with present ED patients continuing to be processed accordingly, albeit delayed, without compromising care.

### Contingency

Sudden onset disasters typically have a finite number of patients involved, although the treating first responders and first receivers are not aware of this as the MCI evolves. When this demand exceeds the daily supply of staff, stuff, and structures due to sheer volume and presenting pace of patients layered on the usual daily census, the conventional MCI response expands. Circumstances such as patient acuity or particular incidents such as burn, blast, or chemical injuries, as well as accompanying familiar blunt or penetrating trauma, now has exceeded the configured space within the structure of the ED. ED staff resilience will seek other structures with space to accommodate the staff and stuff to attend patients within the hospital, approximate to the ED or in the space outside the ED itself. This may have been planned and exercised, or discussed during exercises, to be adapted to meet the demand. If the MCI has not been encountered, exercised, or planned, then ED leadership will discover and adapt as the MCI unfolds: nearby hallways, a family conference room, ED offices that are empty or easily emptied to accept the staff and stuff to attend to an MCI patient. Current ED rooms are repurposed or patients are cohorted,^27^ those pending test results or rides home are placed in nontreatment areas; ED staff coordinates to adapt other treatment structures like the preoperative and postoperative units, or intensive care unit (ICU) level of care rooms. While the MCI efforts are within the ED or have expanded, the current ED and newly arriving ED patients continue to occupy their initial structure, although space may have contracted to continue to deliver functionally equivalent medical care, with minimal increase in risk to the patient.^28^

These strategies are used for the continued rise in the number of patients presenting with COVID-19 symptoms that requires the strict measure to limit exposure. Rooms with doors are a must, personal protective equipment must be available, and the flow of staff has to be managed to limit exposure.

### Crisis

Sudden onset disasters have a finite number of patients concentrated in an initial peak, with the supply of staff and stuff that arrive over time to meet the demand. Most patients can be treated and discharged, while those admitted can be cohorted into space that suits their needs without the strict requirement of standard precautions, unlike the COVID-19 response that will require proper standard precautions. The overall MCI visits will decline over time, MCI hospital admissions will decrease, and the ED volume will eventually resume usual operation levels. This is in distinction to a viral outbreak that will have a gradual onset of those seeking medical attention. While facing an incoming epidemic or pandemic, hospitals can adapt progressively as long as the contagion rates are predictable through estimates that guide stakeholders in the process.^29^

An earthquake in a major metropolitan area could create a catastrophic scenario that would require resources that would have to come from outside the region due to the sheer number of patients. As the COVID-19 pandemic has progressed in each city, the sheer number of patients arriving has been described as “like an earthquake every day”^30^ with an unpredictable increase while there is a lack of inpatient discharges. The resilience of the ED will require a formal process to create the structures with the space to accommodate the COVID-19 oxygen requiring patients, noninvasive ventilators, or ventilators that are accumulating due to a lack of in-patient space.

## T2: SCOPING LITERATURE REVIEW OF CURRENT COVID-19 LITERATURE

The publication of COVID-19 peer-reviewed articles and gray sources is ongoing. This information will demonstrate how hospitals are expanding their surge structure. A literature review was conducted according to the Preferred Reporting Items for Systematic Reviews and Meta-Analyses extension for Scoping Reviews (PRISMA-ScR) checklist,^31^ to include manuscripts published up to 22 March 2020; and if not in print then accepted for online publication ahead of print.

### Search Strategy

**FIGURE 2:**
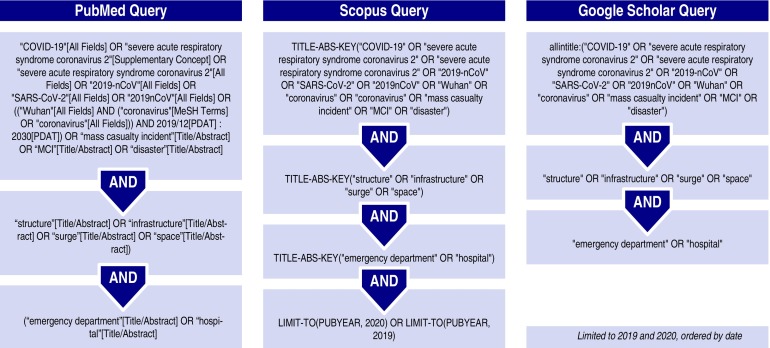
Search Query.

The controlled vocabulary of Medical Subject Headings (MeSH) from PubMed, including subheadings, publication types, and supplementary concepts, was used to identify the entry terms for the search (Figure 2).^32^

The search was performed on PubMed/MEDLINE, Scopus, and Google Scholar from the 15 to 22 March 2020. A search of the gray literature was conducted at the same time.

**FIGURE 3:**
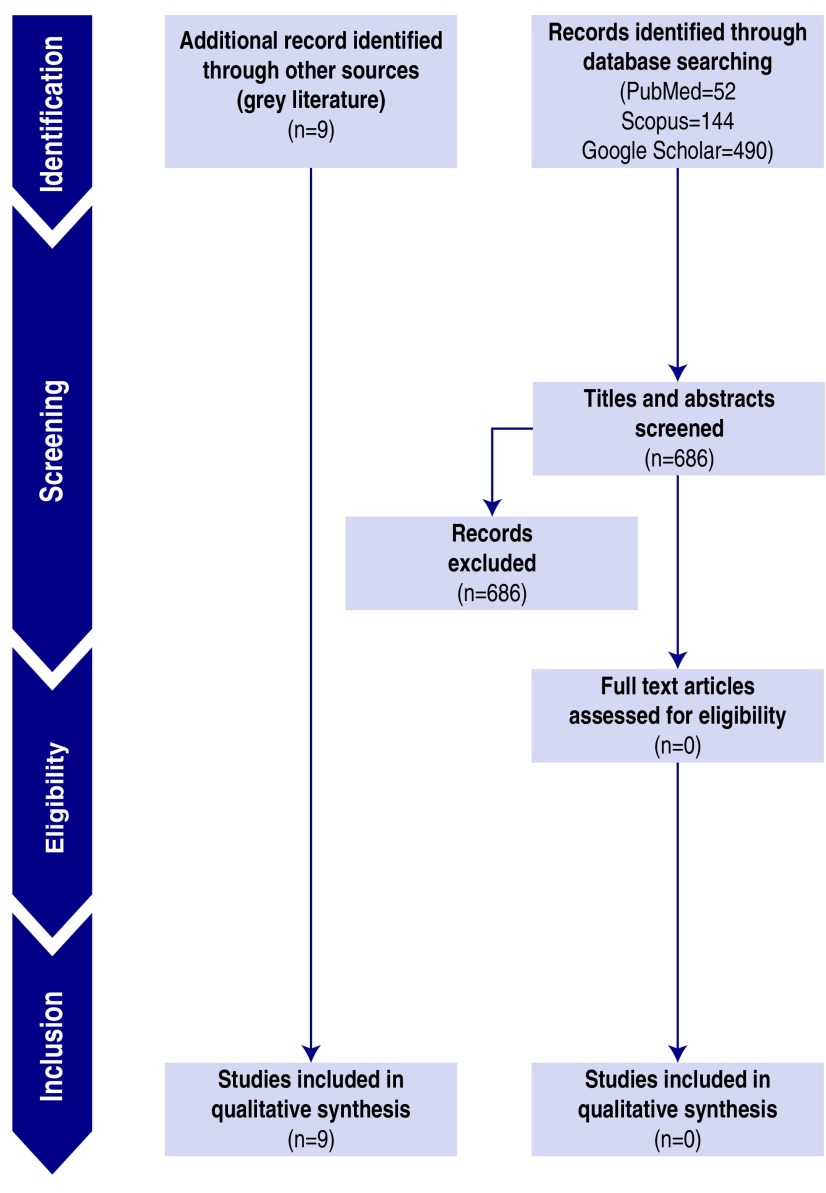
Search Results.

Table 1 and Figure 3 were developed to extract data based on the above review of prior MCI and pandemic surge capacity as discussed earlier.

**TABLE 1:** Search Tool

Team Member	Concern	Mechanism
ED physician	Aerosol	Adapt
ED nurse	Droplet	Repurpose
Respiratory therapy	PPE: store/waste	Cohort
Radiology	PPE: don/doff	Create
Pharmacy	Traffic: patient	
Engineering	Traffic: staff	
IT	Traffic: radiology	
Housekeeping	Traffic: materials	
Logistic/warehouse		
Registration		
Nutrition		
Security		
Administration		

This tool was used to screen literature for relevant content. ED, emergency department; IT, information technology; PPE, personal protective equipment; don, donning (putting); doff, doffing (removing).

Inclusion criteria were as follows: (1) studies describing hospital reaction to the SARS-CoV-2 pandemic and specifically detailing structural changes and infrastructural remodeling to cope with the new challenges imposed by the outbreak; (2) any study design, reports included; and (3) gray literature, also including professional society guidelines, protocols, or consensus statements, peer-reviewed blog posts, and podcasts.

Exclusion criteria were as follows: (1) contents in languages other than English, French, or Italian; and (2) literature without available abstract or full-text.

### Search Findings

Our search did not identify manuscripts to be included in the qualitative analysis. The records identified through gray literature scanning^30,33-41^ are summarized in Table 2.

**TABLE 2:** Scoping Search Results

Title	Source (Journal, Website…)	Team Member/Actor	Concern	Mechanism (How To)
Covid-19 Podcast From Italy With Roberto Cosentini^30^	St. Emlyn’s Blog https://www.stemlynsblog.org/covid-19-podcast-from-italy-with-roberto-cosentini-st-emlyns/	Emergency department physicians	Surge	Split ED into “Lung Diseases Unit” and “Other Diseases Unit”
			Timing for structural modifications	Prepare in time - during the onset phase because presentation rate grow exponentially
			Treatment of COVID-19 patients depending on oxygen and ventilation	Divide the “Lung Diseases Unit” (contaminated path) into a high, medium, and low intensity areas
		Hospital medical direction	Hospital admission increase	Create spaces
COVID-19 Update: An Interview With Andrea Duca, MD^33^	EMplify Emergency Medicine Podcast https://blubrry.com/emplify/57487286/episode-38-covid-19-update-an-interview-with-andrea-duca-md/	Emergency department physicians	Spread of the disease	Create a contaminated/dirty Area and dedicated pathway
			Timing to create dirty area	Progressively adapt to the increasing surge of patients
			Not enough space	Create open space wards and waiting areas where patients are treated while waiting for hospital boarding
				Cohort patients with COVID-19 to gain space
			PPE shortage	Change gloves between patients
			Shortage of equipment and supplies	Rationalize use of equipment and materials
COVID-19: A Powerful Message From Italy^34^	REBEL EM Blog https://rebelem.com/covid-19-a-powerful-message-from-italy/	Emergency department physicians	Spread of the disease	Creation of 2 divided paths inside the ED: a contaminated and a clean one
			Change in respiratory diseases prevalence	Adapt and repurpose spaces to the increasing prevalence, remodeling on the base of oxygen sources and ventilators – dedicating most of the department to see acute respiratory patients. Leave a small clean area of the ED for non-suspect patients
			Timing of changes	Progressively change but expect an exponential increase of presentations so enact changes quickly.
		Hospital medical direction	PPE shortage	Reduce shifts and dedicate personnel to the dirty path to reduce PPE wastage
			Oxygen shortage	Pay attention to oxygen administration
COVID-19 Emergency Department Response Strategies^35^	American College of Emergency Physicians https://www.acep.org/globalassets/new-pdfs/covid-19-for-emergency-department-response-strategies.pdf	Emergency medicine physicians	Risk of contamination/exposure; PPE shortage	Reduce episodes of donning and doffing/patient encounter
				Increasing intercommunication and “door” exam
				Create a screening point outside of the hospital
Duty to Plan: Health Care, Crisis Standards of Care, and Novel Coronavirus SARS-CoV-2^36^	National Academy of Medicine https://nam.edu/duty-to-plan-health-care-crisis-standards-of-care-and-novel-coronavirus-sars-cov-2/	Emergency medicine physicians	Increasing presentation rates	Repurpose current rooms/areas to isolation rooms and infectious care areas
				Cohorting patients
				Boarding, discharge, and admission waiting areas.
National Strategic Plan for Emergency Department Management of Outbreaks Of COVID-19^37^	American College of Emergency Physicians https://www.acep.org/globalassets/sites/acep/media/by-medical-focus/covid-19-national-strategic-plan_0320.pdf	Emergency medicine physicians and hospital medical direction	Spread of disease	Separate areas for patients with respiratory symptoms
				Maximize distance between patients (at least 6 feet)
				Protocols for people accompanying patients in waiting areas and visitors
		Emergency medicine physicians, hospital medical direction, cleaning service	Spread of disease	Protocols for decontamination
		Hospital medical direction	Increased presentation and admission rates	Opening unused areas, patient cohorting, doubling up inpatient rooms
EUSEM Position Paper on Emergency Medical Systems Response to COVID-19^38^	European Society of Emergency Medicine https://eusem.org/news/505-eusem-position-paper-on-emergency-medical-systems-response-to-covid-19	Emergency department	Spread of the disease	Increase number of isolation rooms, possibly with negative pressure and adequate ventilation
				Favor physical barriers between patients
				Specific triage areas for suspect patients
				Create patient dedicated waiting areas for suspects before boarding
				Minimization of movements for suspect patients (ED, radiology, bathrooms)
				Cohorting of patients is reasonable
				Maintain a distance of at least 1 meter
				Don’t allow visitors or co-operators in the area
		Cleaning service	Spread of the disease	Specific decontamination protocols
				Treat waste from the contaminated area as a high risk biological material
		Emergency department	Increased presentation rate	Limit access to ED to patients with severe symptoms. Asymptomatic or mild symptomatic patients should seek advice to GP
			PPE shortage	Increase stockpiles and supplies
COVID-19 First Line – 10 Topics From the EDs During Corona^39^ *(Prima Linea COVID-19 - 10 cose… dai PS ai tempi del Corona)* COVID-19 First Line Report – Organizational Structure of EDs Before and During the Outbreak^40^ *(Rapporto prima linea COVID-19 - Assetto organizzativo gestionale dei PS/DEA nell’ambito di focolaio epidemico o pre-epidemico)*	Italian Society of Emergency Medicine *(Società Italiana Medicina d’Emergenza-Urgenza)* https://www.simeu.it/w/articoli/leggiArticolo/3992/leggi https://www.simeu.it/w/articoli/leggiArticolo/3964/leggi	Emergency department	Increased presentation rate	Prepare to the surge at least 10 days before
				Repurpose areas increasing possibility of ventilatory support
			Spread of the disease	Reduce visits and activities to essentials
				Create “filter zones” (forward triage or screening areas)
				Separate Areas for Suspect infectious patients
			PPE shortage	Increase stockpiles and supplies
			Oxygen shortage	Increase stockpiles and supplies
Checklist for Healthcare Facilities: Strategies for Optimizing the Supply of N95 Respirators during the COVID-19 Response^41^	Centers for Disease Control and Prevention https://www.cdc.gov/coronavirus/2019-ncov/hcp/checklist-n95-strategy.html	Emergency Department	Spread of the disease, PPE shortage	Use isolation rooms
				Use physical barriers between patients
				Maintain adequate ventilation

The retrieved materials were mostly found on websites of national and international professional societies. Of note, 2 podcasts^30,33^ and 1 blog post^34^ were found reporting structural adaptations of the ED of the hospital of Bergamo, Italy.

Sources wrote of the need to create dedicated pathways to divide potentially infectious patients (COVID-19) the others (Clean) presenting to the ED. Among the other suggestions made, the management of spaces should take into account the increased prevalence of the disease, thus requiring a track dedicated to respiratory symptoms (reported as approximately 90% of current ED activity in Bergamo hospital).^30^ Physical barriers should actively separate the 2 fluxes of patients.

The 2 articles are pending online publication ahead of print in the *Disaster Medicine and Public Health Preparedness* journal (personal communication). Medicine relies on tradition, and this is evident in the observation of Faccincani^42^ of his experience in Milan. Treatment of hemorrhagic shock calls for transfusion of “2 units of packed red blood cells and tell the blood bank to stay 2 units ahead” theory applies to his experience to stay 1 ventilator ahead. Extending this to the awareness of when to start seeking more structure for treatment space is when you are comfortable to be aware of the period of time that would require a structure to be adapted, repurposed, or created. The number of references that assign number of patients to be prepared to treat during the timeline of surge, from conventional to contingency to crisis, in peer-reviewed literature, professional societies, nongovernment and government publications is far too great to cite. Only a hospital that is studying their trends can determine when to begin the reconfiguration of a structure. The caution to stay “1 structure ahead” seems to be prudent. When the team has identified structures within their facility to reconfigure, the time that this would take is part of the calculus when prioritizing when to expand the ED COVID-19 footprint.

Gagliano et al.^43^ wrote of their experience in the Northern City of Lodi, the epicenter of the pandemic in Italy. Their management team relied on accurate data to guide their surge response from the first recognition that the SARS-CoV-2 virus was in the community as COVID-19 patients began to appear. Their conventional management was to cohort patients based on their oxygen requirements and potential for aerosol (noninvasive ventilator treatment) as well as those placed on ventilators to maximize similar structure and space. As the ICU structure was filling up, they turned to the operative theater to increase their ventilator and monitoring space after the first days. While this required minimal investment of resources to reconfigure to limit exposure to staff and to have sufficient supplies, the team was identifying structures that could be adapted, repurposed with little creation to have space to manage the ventilator-dependent patients. The ward (structure) identified already had monitoring capability with the approach to create a filter zone between contaminated and noncontaminated spaces, increased warehouse (stuff), and sanitary (environmental services/housekeeping) space. By the 8th day, they had completed the transformation with cohort structures with space for oxygen-dependent patients.

## T3 = CREATION OF A CHECKLIST FOR COVID-19 SURGE STRUCTURE PLANNERS

Through a review of prior MCI surge capacity literature, this study seeks to find guidance that can be used to understand the mechanisms^44^ to provide the structures necessary to meet the demands of the COVID-19 pandemic (Table 1). The creation of an expert team^28^ to provide critical infrastructure recommendations beyond the scope of the treatment team is imperative. Once the assembled team (Table 1) understands the likely timeline of the COVID-19 patient load demands on their hospital from conventional to contingency to crisis, they can grasp the challenges understanding the limitation of their structure to provide the necessary space to treat these patients while limiting exposure to staff while treating other patients and limiting their exposure.

This process begins with an agreement on how to create a single path *into* the ED. Saturation messaging in the community is crucial to prevent the ED from becoming a vector, overwhelmed with patients that do not require a detailed evaluation. The external tent structures now ubiquitous outside Italian EDs have become a fundamental screening space. This study does not address the creation or deployment of these or any other external structure or alternate care sites.

The team has to account for exposure by means of droplet from the patient’s arrival in the parking lot. Patients should notify staff of their arrival while still in the car to obtain a surgical mask or to use another form of barrier over the face to limit droplets. Space for this staff can be created in or near the ED entrance. The team can do a walk-through as if they were the patient approaching the ED to understand where signage should be placed to direct the patient on a *COVID-19 path* in distinction from the *“Clean” patient path*, or all other patients. This can be done with poles and ropes if available or tape on the floor with arrows, especially if either the Clean or COVID-19 areas are not in the usual ED location in the hospital. Volunteers wearing masks or facial barriers that are more than 6 feet from either path can direct the patient to their treatment destination. Space for volunteers will require consideration if that is outside the entrance or within the structure of the ED to ensure they can manage their activities.

Typically, the ED waiting space is crowded with patients who are waiting to be assessed in triage, have been triaged and waiting to be examined, or waiting for results of tests or images ordered at triage. These patients are usually not sequestered or removed from patient family members. Now the team has to create a COVID-19 waiting space *separate and distinct* from the Clean waiting space. A distance of at least 6 feet from any COVID-19 family member should be ensured, because they may have been exposed and able to transmit the SARS-CoV-2 virus, and preferably not even close to any Clean patients or family members. The COVID-19 space should be well ventilated and in proximity to staff that can monitor those patients waiting to go to the ED treatment space. Some EDs feature vending machines and televisions to occupy those waiting, and the team will have to address the allocation and maintenance of these in relationship to the prior adapted, repurposed, or created triage waiting area structure or space. Registration of COVID-19 patients should be separate and distinct from Clean patients. This can be accomplished in the prior registration space or space that has been adapted, repurposed, or created to accommodate a computer desktop, a portable device on wheels, or a tablet for the registration personnel.

The depth and breadth of treating COVID-19 patients requires oxygen, compressed medical air, and vacuum for suction.^45^ Outlets and piping may be hidden behind walls created when space was repurposed to become an office, conference room, or other nontreatment areas. Ventilators and other machines may require different electrical outlets or local grids to avoid overloading any circuits. The treatment team members, physicians, and nurses are encouraged to discuss with the engineer team member the specific vital components of a reconfiguration.

Similarly, contaminated solid and fluid waste, as well as contaminated laundry and other trash, will require proper positioning in structures or spaces that were not designed for the sophisticated support required to treat the COVID-19 patients. The team’s attention to this detail will provide the opportunity to maintain a secure infection control loop. Environmental services or housekeeping standard operating procedures during COVID-19 are essential with droplet control paramount to reduce exposure to all as well as rapid turnaround of space for the next patient. They will require pathways from their base of operations through the structure to their duty location with their advanced materials with the need to resupply at a location that may be closer than their base. Because these supplies may be of limited stock and valued by many, a secure location in the structure is required.

Central monitors may be able to be moved from 1 structure to another to create adapted repurposed or created space. This reconfigured space will have to accept computers or charging stations for portable electronic health record devices. The information technology and engineering team members may have innovative solutions to deploy these support assets with minimal cost and time to deploy, in safe and convenient locations.

The path that a patient takes from their ED space, with a door if available, unless in a cohort of similar patients, to radiology or their in-patient destination should be clearly marked as COVID-19 patients, with plastic or other barriers to prevent aerosol droplets landing on Clean hallways, walls, or doors along the way. The creation of filter zones, as mentioned by the team in Lodi using available construction plastic or plastic strips similar to the barriers in refrigeration compartments of stores that hang from the ceiling to the floor, provide a droplet barrier. Ideally, these can be obtained and stored before the need with anchor points along the locations in the structure.

Portable imaging machines are cumbersome and to be factored into the process of surge structure design. Also, beds, gurneys, and the associated IV poles, tables, and other materials that are required for a patient in the treatment space are to be accounted. One may best be served to take a gurney or bed with you to a structure that was not designed for in-patient treatment, such as a clinic or IV infusion center where there are only incline chairs or exam tables, to see if these patient support devices can maneuver in the tight spaces.

Most structures within a hospital have a just-in-time process to manage stuff to maintain organization, as a means to know what is used to restock and to charge the patient. In a pandemic, this will likely not be entirely possible to implement, although both the storage and resupply have to remain convenient, secure, and free from contamination unless approximate as necessary to maximize patient treatment. The pharmacy storage and distribution process will rapidly outpace any automated medication dispensing system (eg, BD Pyxis^46^). The team’s reliance on the guidance of the pharmacy and nursing representatives will be necessary to assure an efficient loop for the treatment team to have what they need in the reconfigured structure.

## T4 = COLLECTION OF COMPLETED CHECKLISTS TO IMPROVE THE COVID-19 SURGE STRUCTURE PLANNING

There is no argument that the evidence-based medicine approach using the Delphi method would have been in line with prior research in the field.^27,28,36,38,39^ These are extraordinary times, and the authors accept this limitation to produce a checklist to guide the team that has been tasked to reconfigure their structure for their surge COVID-19 response. The translational science intention is to create a checklist guide where none existed previously, fully aware that this checklist has not been vetted or verified or tested through simulation or live exercise. Translational science calls for feedback using the checklist (Table 3; an editable version is available as Online Supplemental Material 1) for the authors to continue this study to use to perform the T2 PRISMA review after a COVID-19 research body of work has been published on this topic. The checklist is to be used to collaborate with all relevant actors in real-time as the pandemic is unfolding to maximize the surge structure response in a timely manner, with minimal disruption to the non–COVID-19 patients in the facility, using available staff and stuff with the intent to return the structure to the prior state as soon as possible to resume the activities before this pandemic.

**TABLE 3:** Surge Structure Checklists

TEAM MEMBER		NAME		CELL		EMAIL	
ED PHYSICIAN							
ED NURSE							
RESPIRATORY THERAPY							
RADIOLOGY							
PHARMACY							
ENGINEERING							
INFORMATION TECH. (IT)							
HOUSEKEEPING							
LOGISTIC/WAREHOUSE							
REGISTRATION							
NUTRITION							
SECURITY							
ADMINISTRATION							

STRUCTURE	MECHANISM				Person in Charge	Time to Change	Distance to ED
	ADAPT	REPURPOSE	COHORT	CREATE			
							
							
							
							

CONCERN	Person in Charge	ONGOING	ADDRESSED	N/A		TASKS/NOTES	
AEROSOL (DOOR?)							
DROPLET (filter zone)							
PPE: STORE/WASTE							
PPE: DON/DOFF							
TRAFFIC: PATIENT							
TRAFFIC: STAFF							
TRAFFIC: RADIOLOGY							
TRAFFIC: MATERIALS							
BED/STRETCHER/CHAIR							
POLES							
TABLE							
ELECTRIC							
GASES							
WATER/TRASH							
VENT/NIV							

## Supplementary material

For supplementary material accompanying this paper visit http://dx.doi.org/10.1017/dmp.2020.57. click here to view supplementary material
