# Supplementary material for: Translating COVID-19 Pandemic Surge Theory to Practice in the Emergency Department: How to Expand Structure
Source: Disaster Med Public Health Prep. 2020 Mar 27:1–10. doi: 10.1017/dmp.2020.57 (PMC7156581; doi:10.1017/dmp.2020.57)
Supplement: Supplementary file 1 [file S1935789320000579sup001.docx]

**Table 3**: Surge Structure Checklists

| **TEAM MEMBER** | **NAME** | **CELL** | **EMAIL** |
| --- | --- | --- | --- |
| ED PHYSICIAN |  |  |  |
| ED NURSE |  |  |  |
| RESPIRATORY THERAPY |  |  |  |
| RADIOLOGY |  |  |  |
| PHARMACY |  |  |  |
| ENGINEERING |  |  |  |
| INFORMATION TECH. (IT) |  |  |  |
| HOUSEKEEPING |  |  |  |
| LOGISTIC/WAREHOUSE |  |  |  |
| REGISTRATION |  |  |  |
| NUTRITION |  |  |  |
| SECURITY |  |  |  |
| ADMINISTRATION |  |  |  |

| **STRUCTURE** | **MECHANISM** | | | | Person in Charge | Time to change | Distance to ED |
| --- | --- | --- | --- | --- | --- | --- | --- |
|  | ADAPT | REPURPOSE | COHORT | CREATE |  |  |  |
|  |  |  |  |  |  |  |  |
|  |  |  |  |  |  |  |  |
|  |  |  |  |  |  |  |  |
|  |  |  |  |  |  |  |  |

| **CONCERN** | **Person in Charge** | **ONGOING** | **ADDRESSED** | **N/A** | **TASKS/NOTES** |
| --- | --- | --- | --- | --- | --- |
| AEROSOL (DOOR?) |  |  |  |  |  |
| DROPLET (filter zone) |  |  |  |  |  |
| PPE: STORE/WASTE |  |  |  |  |  |
| PPE: DON/DOFF |  |  |  |  |  |
| TRAFFIC: PATIENT |  |  |  |  |  |
| TRAFFIC: STAFF |  |  |  |  |  |
| TRAFFIC: RADIOLOGY |  |  |  |  |  |
| TRAFFIC: MATERIALS |  |  |  |  |  |
| BED/STRETCHER/CHAIR |  |  |  |  |  |
| POLES |  |  |  |  |  |
| TABLE |  |  |  |  |  |
| ELECTRIC |  |  |  |  |  |
| GASES |  |  |  |  |  |
| WATER/TRASH |  |  |  |  |  |
| VENT/NIV |  |  |  |  |  |
